# Supplementary figures and images for: Same but not alike: Structure, flexibility and energetics of domains in multi-domain proteins are influenced by the presence of other domains
Source: PLoS Comput Biol. 2018 Feb 12;14(2):e1006008. doi: 10.1371/journal.pcbi.1006008 (PMC5825166; doi:10.1371/journal.pcbi.1006008)

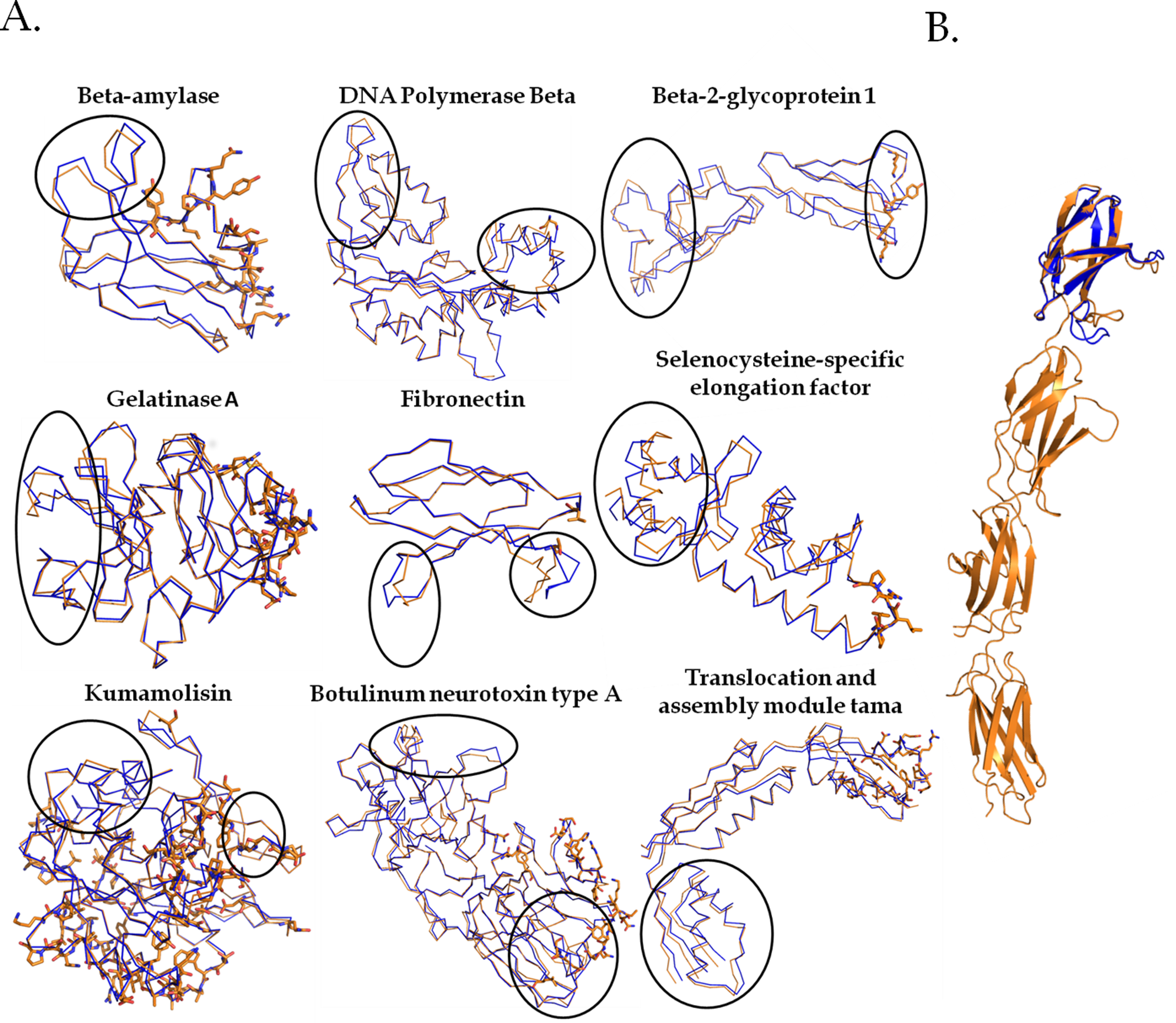

Supplement: S1 Fig — (A) 9 pairs out of 20 protein domain pairs show structural deviations at regions other than domain-domain interface residues. Such regions are encircled in black while the domain-domain interface residues are represented in sticks. These deviations were observed irrespective of the number of domain-domain interface residues. (B) Cartoon representation of fibronectin (MD is colored in bright orange and ID is colored in blue). The domains in fibronectin are observed to be non-interacting. (TIF) [file pcbi.1006008.s001.tif]

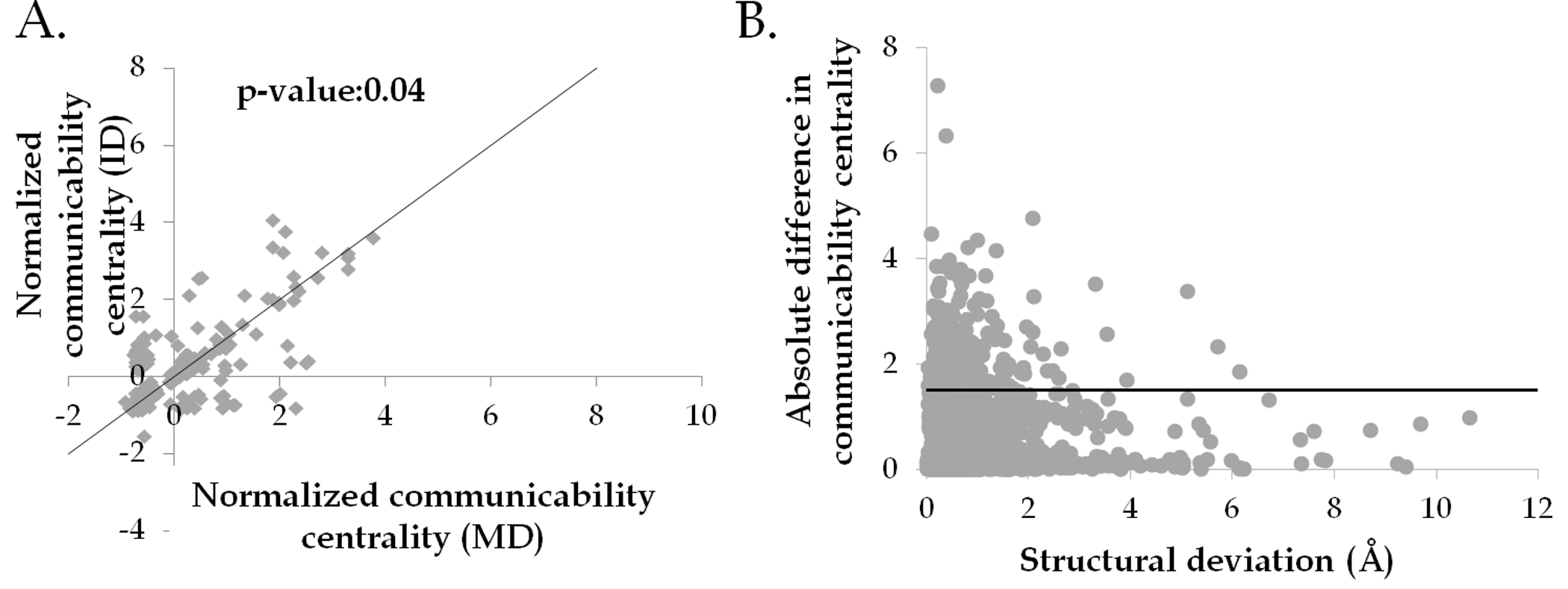

Supplement: S2 Fig — (A) Normalized coc distribution for the functional residues. The X-axis represents the coc of MD and the Y-axis represents the coc of ID. The solid line in the plot represents the unity line. (B) Scatter plot of the absolute difference of coc of MD and ID and the structural deviation between corresponding Cα. The solid line is drawn at the absolute difference of coc of 1.5. Values above 1.5 signify the significant difference between coc of MD and ID. (TIF) [file pcbi.1006008.s002.tif]

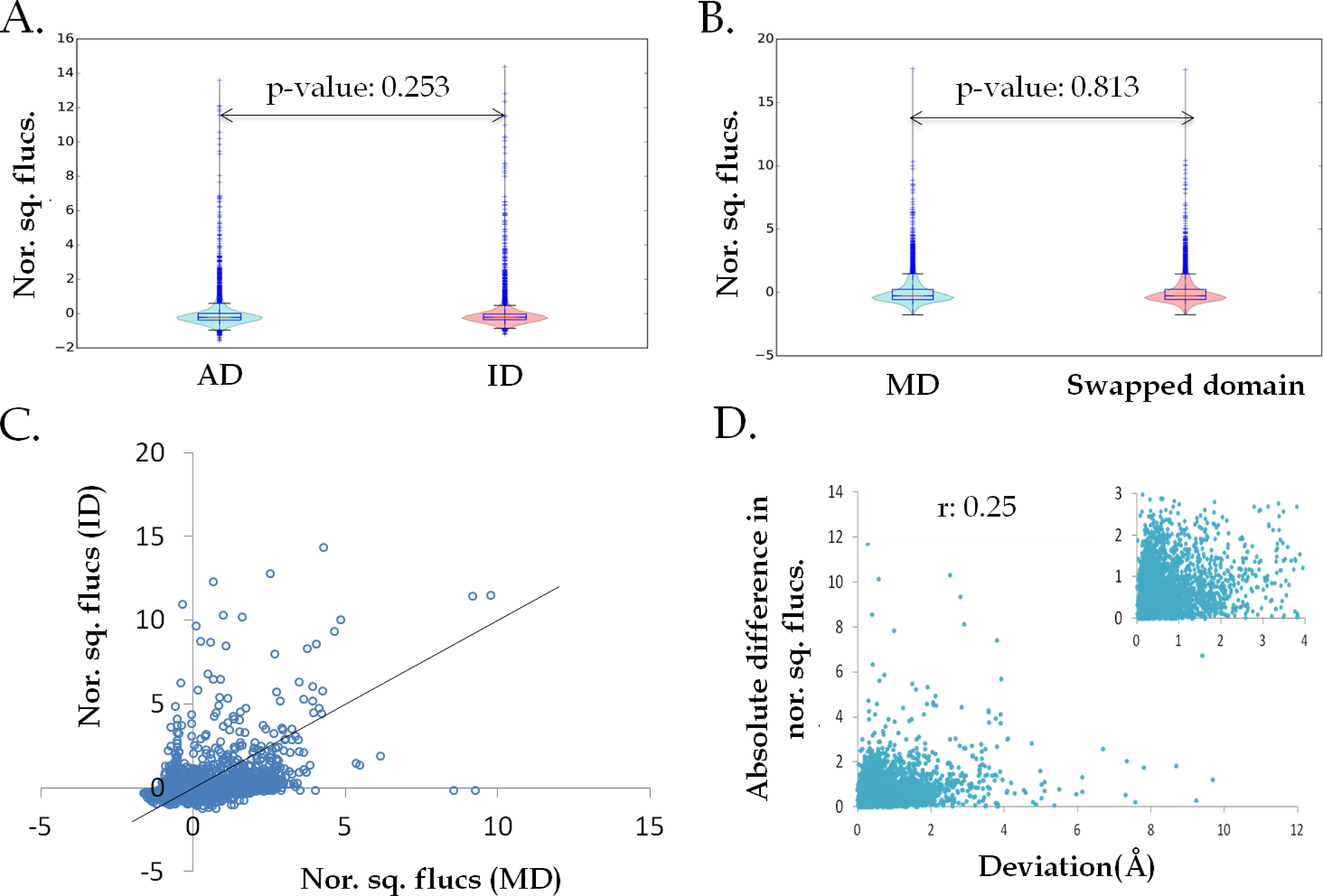

Supplement: S3 Fig — (A) Distribution of normalized square fluctuations of ID and the control dataset 2 (AD). The two distributions are similar (two-sample KS test, p-value: 0.253). (B) Distribution of normalized square fluctuations of MD and the control dataset 3 (Swapped domain). The two distributions are similar (two-sample KS test, p-value: 0.813). (C) Scatter plot of the normalized square fluctuation of all the non-functional and non-interface residues, The X-axis represents the normalized square fluctuation for MD and the Y-axis represents the normalized square fluctuation for ID. The solid line in the plot represents the unity line. (D) Distribution of absolute difference in normalized square fluctuation and the structural deviation between corresponding Cα. Spearman correlation coefficient for the distribution is 0.25. The inset shows the expanded view of the dense region. (TIF) [file pcbi.1006008.s003.tif]

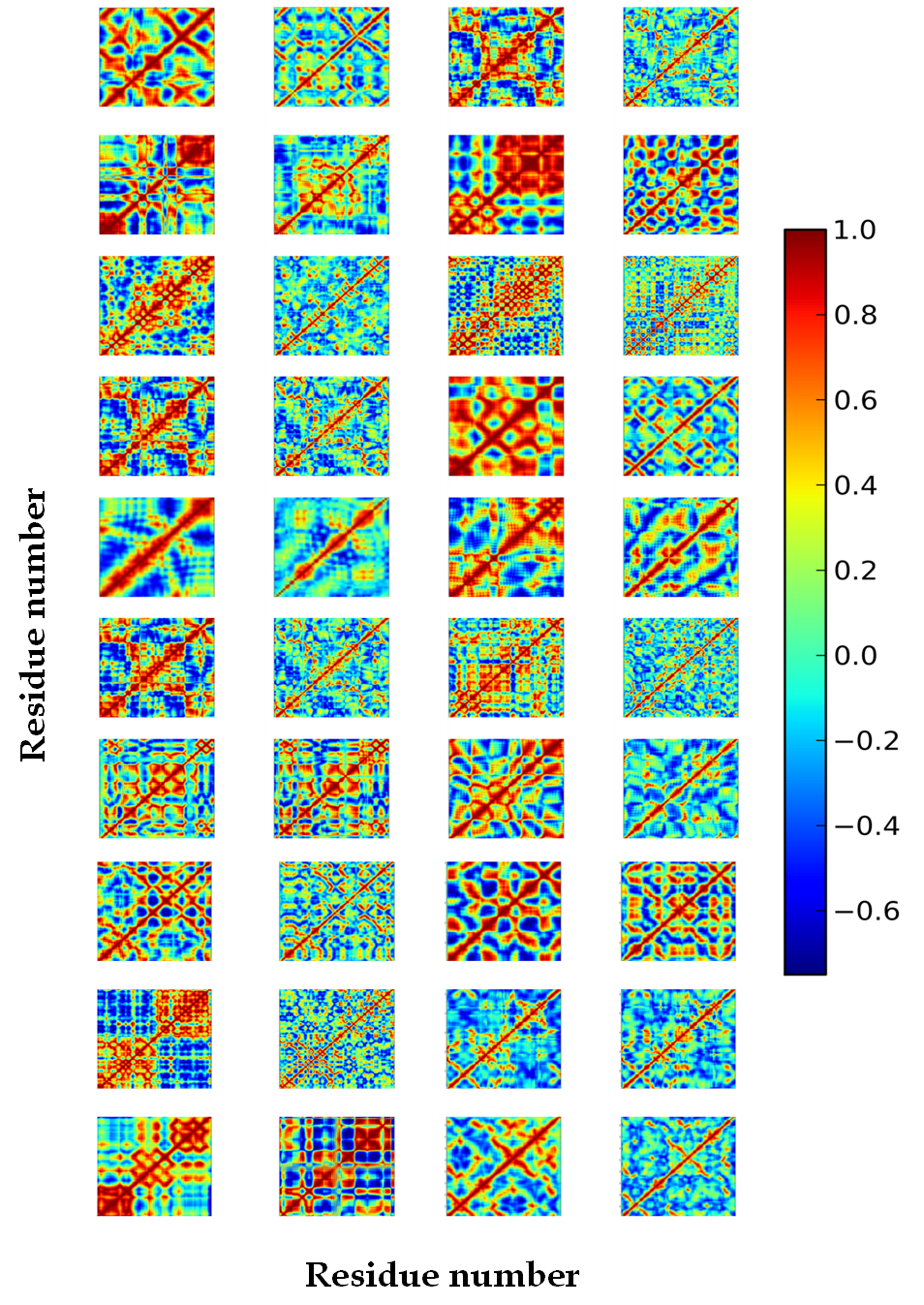

Supplement: S4 Fig — The first and the third column represent the cross-correlation matrices for MD in domain pairs. The second and the fourth column represent the cross-correlation matrices for the corresponding ID of domain pairs. The residues have been observed to be tightly dynamically coupled in MD as compared to ID. The color bar represents the cross-correlation value. The X-axis and the Y-axis represent the residue number. (TIF) [file pcbi.1006008.s004.tif]

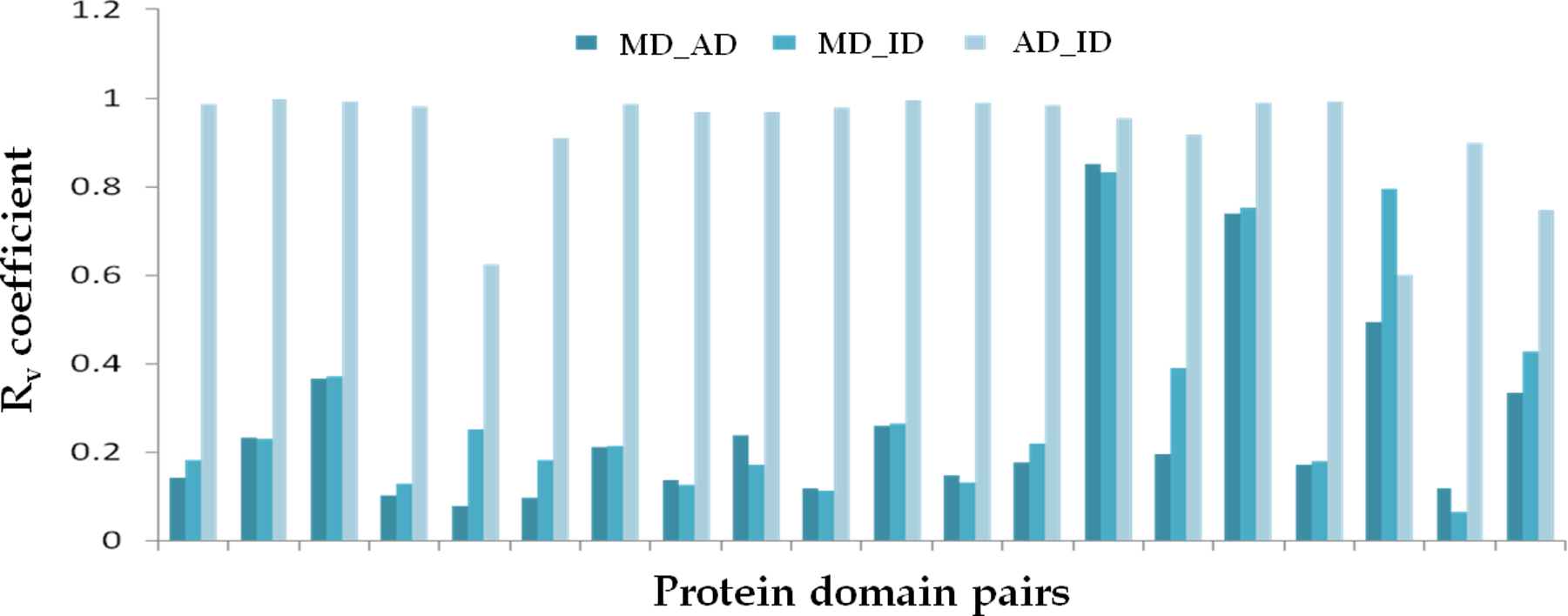

Supplement: S5 Fig — For all the domain pairs, three Rv coefficients have been plotted. Rv coefficients have been calculated for cross-correlation matrices of MD and the control dataset 2 (AD), cross-correlation matrices of MD and ID, and cross-correlation matrices of ID and the control dataset 2 (AD). The high Rv coefficient for ID and SD implies that the low Rv coefficients observe for MD and ID are not a manifestation of crystallization artefact. (TIF) [file pcbi.1006008.s005.tif]

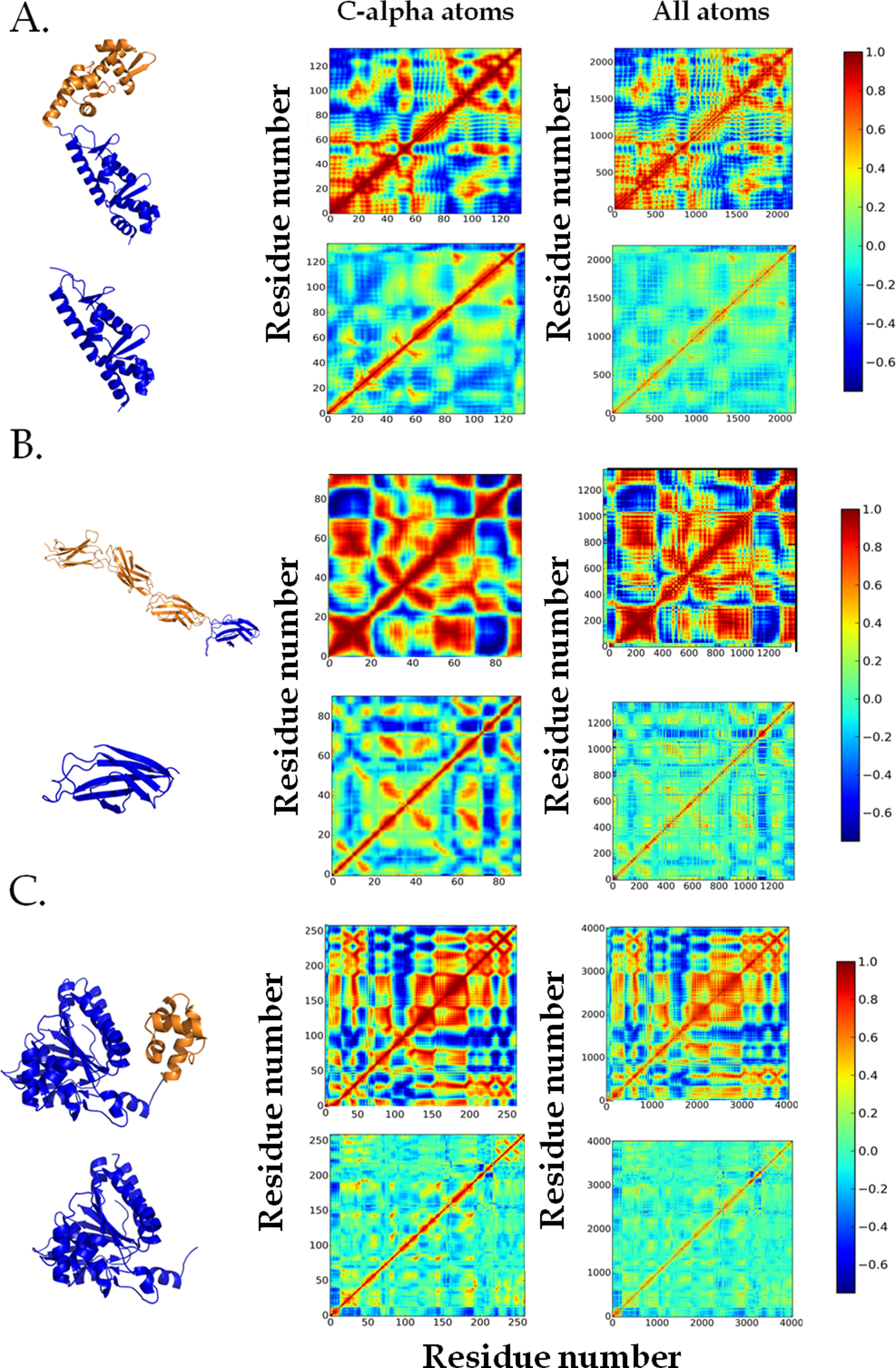

Supplement: S6 Fig — For three domain pairs, molecular dynamic simulations were carried out for 400 ns. (A) Selenocysteine elongation factor (B) Fibronectin (C) DNA repair and recombination protein radA protein. Cartoon representations of the domain pairs are shown on the extreme left, with common domain colored blue. The left side matrices represent the cross-correlation of the residues at C-alpha level and the right matrices represent the cross-correlation at the all-atom level. The first rows in (A, B, and C) represent MD and the second rows in (A, B, and C) represent ID. The color bars represent the cross-correlation value. (TIF) [file pcbi.1006008.s006.tif]

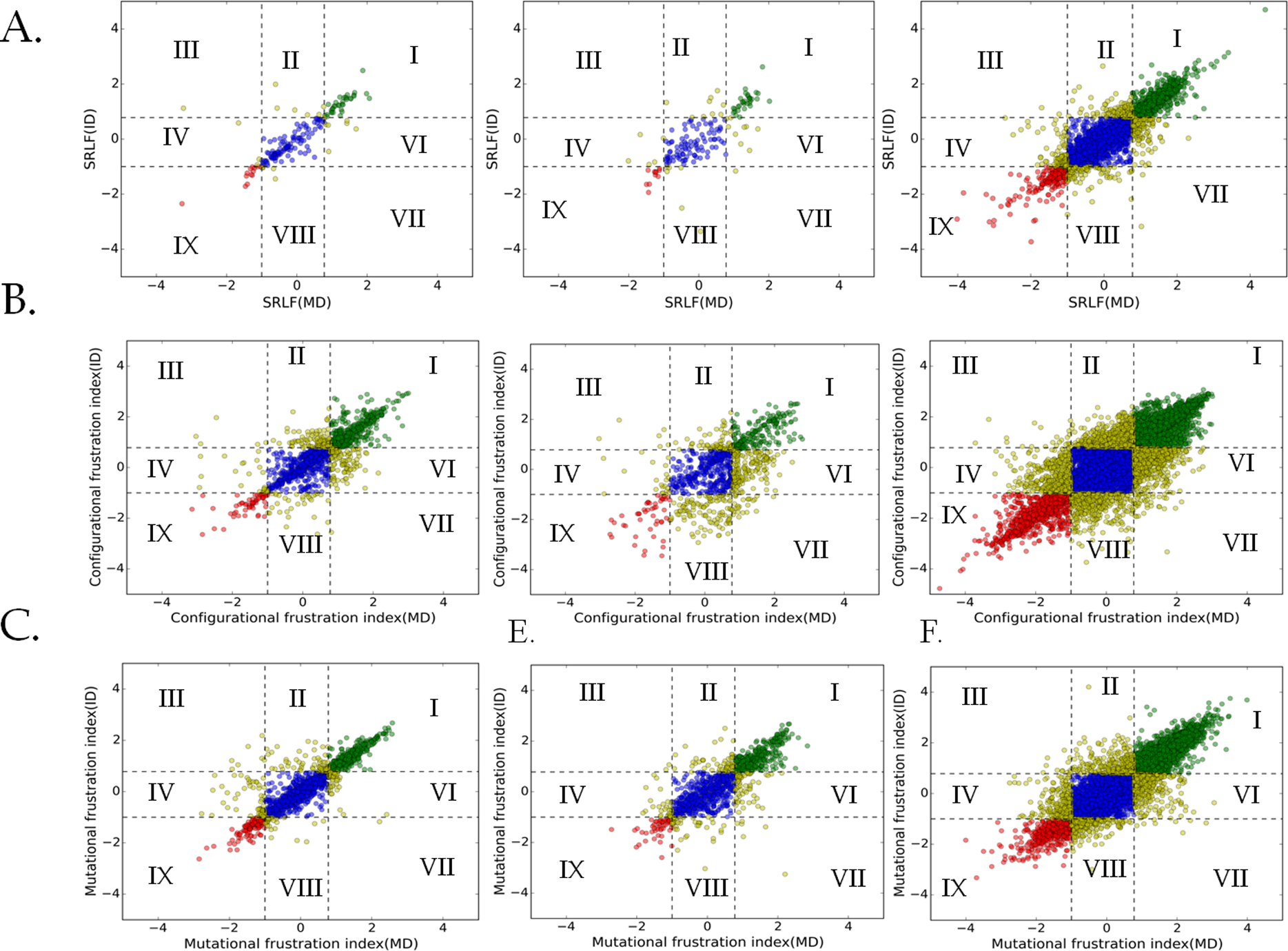

Supplement: S7 Fig — Scatter plot of the distribution of (A) single residue level frustration (SRLF) (B) configurational frustration index and (C) mutational frustration index. The left panel represents functional residues, centre panel represents interface residues and the right panel represents non-interface and non-functional residues for all domain pairs. The X-axis represents the frustration index for MD and the Y-axis represents the frustration index for ID. Residues which are minimally frustrated (MF) in both MD and ID are represented as a green filled circle, residues which are neutrally frustrated (NF) in both MD and ID are represented as blue filled circle and residues which are highly frustrated (HF) in both MD and ID are represented as red filled circles. The residues showing the difference in the type of frustration between MD and ID are represented as a yellow filled circle. The dotted lines have been drawn to represent the cut-off used for the definition of frustration index (MF: FI ≥ 0.78; HF: FI ≤ -1 and NF: -1 < NF < 0.78). (TIF) [file pcbi.1006008.s007.tif]

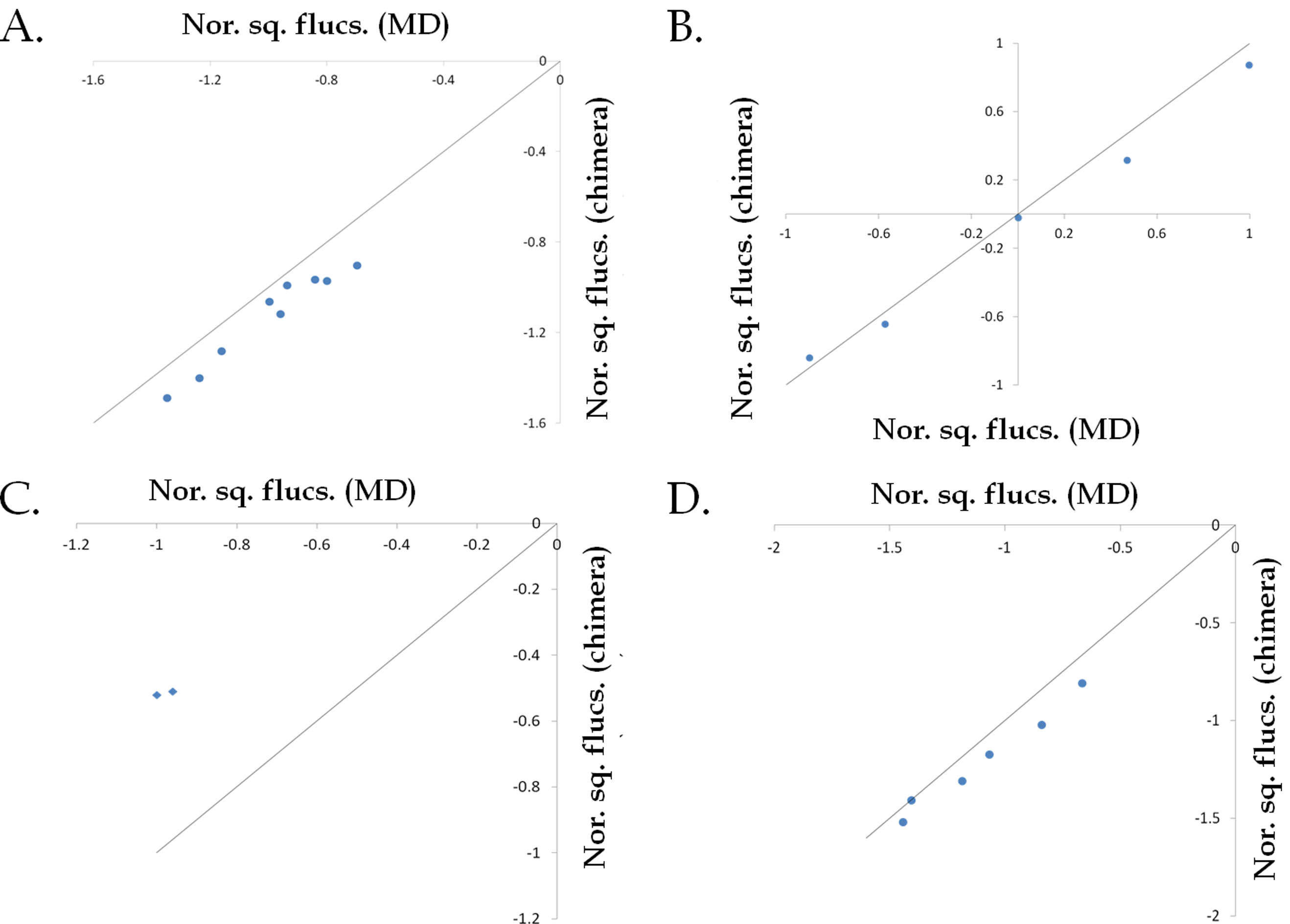

Supplement: S8 Fig — Scatter plots for functional residues of (A) phosphoribosylanthranilate isomerase, (B) cyclophilin, (C) sialidase and, (D) hexokinase-1. The X-axis represents the normalized square fluctuation for multi-domain protein (MD) and the Y-axis represents the normalized square fluctuation for chimera protein. The solid line in all the plots represents the unity line. (TIF) [file pcbi.1006008.s008.tif]

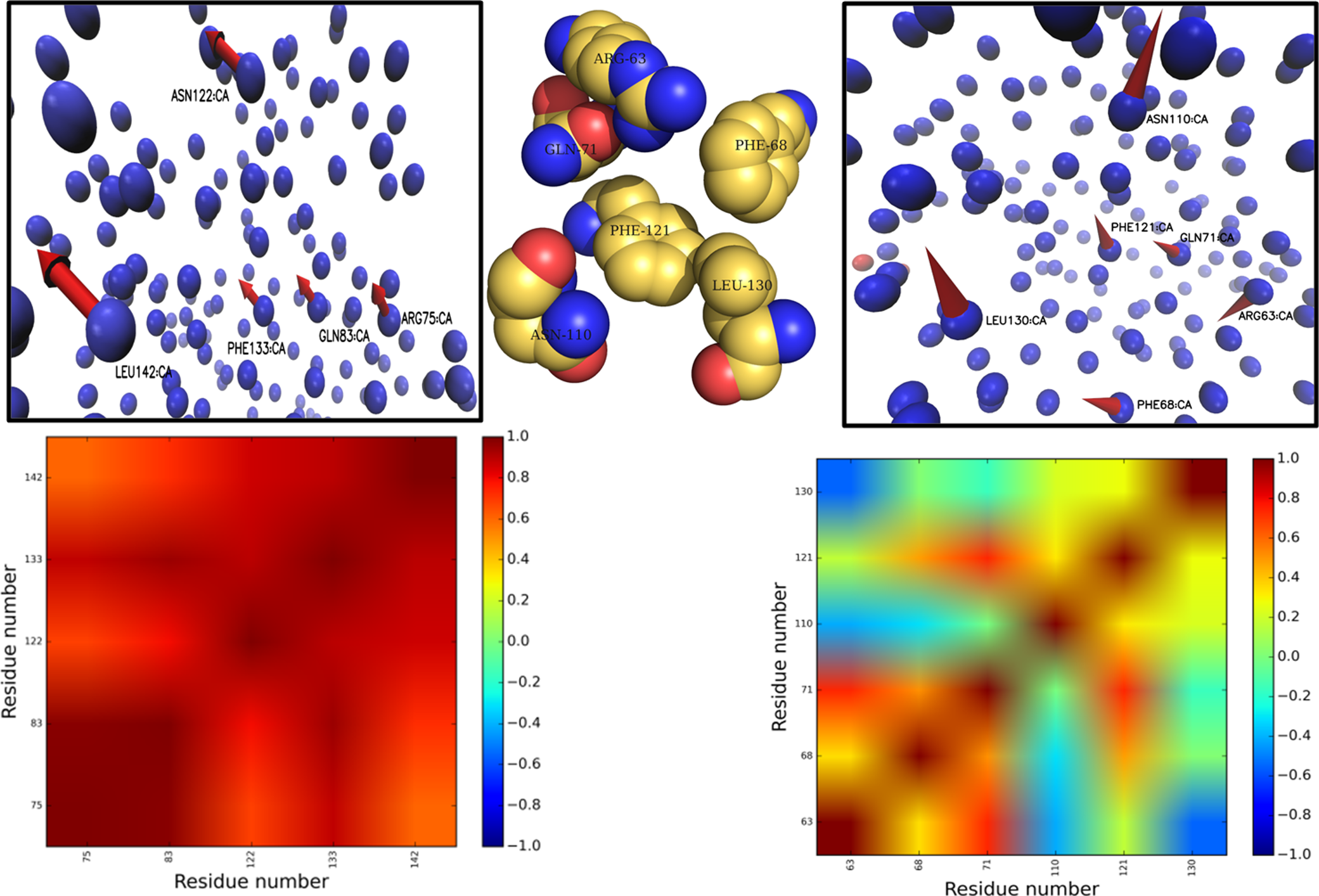

Supplement: S9 Fig — In the upper left panel and the upper right panel, the Cα residues are represented as blue spheres and the directionality and the magnitude of the first mode of motion for functional residues is represented by a red arrow. Longer the arrow higher is the magnitude of the motion. The upper centre panel represents the space-fill representation of the functional residues (cyclosporin binding pocket). The lower left and right panel represents the cross-correlation matrices for functional residues for multi-domain and the single-domain protein respectively. The pocket closing movement of the protein in single-domain protein is superseded by the global domain movement. (TIF) [file pcbi.1006008.s009.tif]

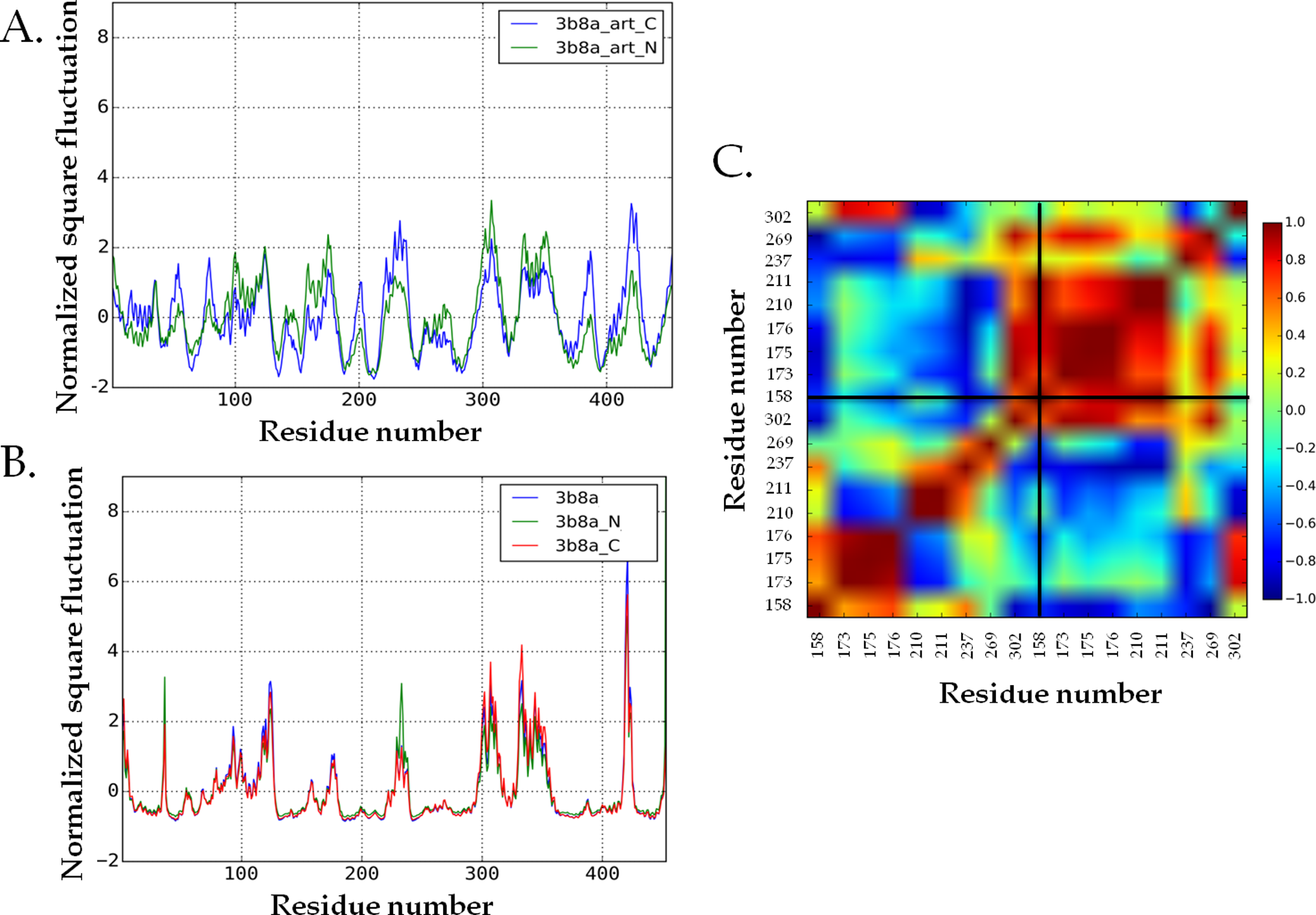

Supplement: S10 Fig — (A) Distribution of normalized square fluctuation of the N-terminal (3b8a_art_N) and C-terminal (3b8a_art_C) of the chimeric hexokinase-1. Certain residues show differences in the flexibility between the two domains. (B) Distribution of normalized square fluctuation of the full-length yeast hexokinase-1 (3b8a), yeast hexokinase-1 with 9 residues removed from the N-terminal (3b8a_N) and yeast hexokinase-1 with 9 residues removed from the C-terminal (3b8a_C). The distributions are highly similar, suggesting that the amino-acids deletions do not affect the flexibility profile. (C) Cross-correlation plot of the functional residues of chimeric proteins. The numbering of the functional residues has been kept same for the N-terminal and the C-terminal domain. The solid line separates the X-axis and the Y-axis into the N-terminal and the C-terminal domain. The X-axis and the Y-axis represent the residue number of the functional residues. (TIF) [file pcbi.1006008.s010.tif]
