# Supplementary material for: Same but not alike: Structure, flexibility and energetics of domains in multi-domain proteins are influenced by the presence of other domains
Source: PLoS Comput Biol. 2018 Feb 12;14(2):e1006008. doi: 10.1371/journal.pcbi.1006008 (PMC5825166; doi:10.1371/journal.pcbi.1006008)
Supplement: S1 Text — Table A. SCOP fold annotations, number of interface residues, length of the linker, size of the domain and the nature of differences observed for the identical domain pair dataset. Table B. SCOP fold annotations, number of interface residues, length of the linker, size of the domain and the nature of differences observed for the homologous domain pair dataset. Table C. PDB codes and macromolecule name of pairs of same proteins used for the analysis with their respective resolution (Å). Table D. PDB codes of homologous domains pairs with their resolution (Å). (DOCX) [file pcbi.1006008.s011.docx]

**Table A.** SCOP fold annotations, length of the interface residues, length of the linker, size of the domain and the nature of differences observed for the identical domain pair dataset.

| PDB id (MD) | PDB id (ID) | Common domain directionality | SCOP fold (common domain) | SCOP fold (tethered domain) | No. of interface residues | Length of the linker | Size of the domain^#^ | Nature of difference observed (S/R/D/E)^##^ |
| --- | --- | --- | --- | --- | --- | --- | --- | --- |
| 1B90 | 1CQY | C-terminal | b.3 | c.1 | 27 | 6 | 417; **99** | S/R/D/E |
| 1BLF | 1NKX | C-terminal | c.94 | c.94 | 24 | 11 | 329; **356** | D/E |
| 1BPD | 1RPL | C-terminal | a.60,d.128 | d.218 | 2 | 5 | 83; **57**; **187** | S/R/D/E |
| 1C1Z | 4JHS | C-terminal | g.18,g.18 | g.18,g.18,g.18 | 5 | 5 | 62; 58; 63; **60**; **87** | S/D/E |
| 1CK7 | 1RTG | C-terminal | b.66 | a.20,d.92,g.14,g.14,g.14 | 22 | 9 | 77; 109; 61; 68; 78; **200** | S/R/D/E |
| 1FNF | 1FNA | C-terminal | b.1 | b.1,b.1,b.1 | 4 | 7 | 94; 91; 99; **84** | S/R/D/E |
| 1T1E | 1GTG | C-terminal | c.41 | d.58 | 95 | 6 | 179; **355** | S/D/E |
| 1T4G | 2GDJ | C-terminal | c.37 | a.60 | 4 | 5 | 60; **278** | D/E |
| 1XTI | 1T5I | C-terminal | c.37 | c.37 | 6 | 8 | 209; **169** | S/R/D/E |
| 3BTA | 3FUO | C-terminal | b.29,b.42 | d.92,h.4 | 30 | 7 | 546; 335; **207; 217** | S/R/D/E |
| 4GCR | 1DSL | C-terminal | b.11 | b.11 | 28 | 6 | 85; **89** | D/E |
| 1EUT | 1EUR | N-terminal | b.68 | b.1,b.18 | 7 | 5 | **356**; 103; 142 | D/E |
| 1FCK | 1LCT | N-terminal | c.94 | c.94 | 24 | 7 | 334; **337** | R/D/E |
| 1GV2 | 1GV5 | N-terminal | a.4 | a.4 | 5 | 6 | 55; **47** | R/D/E |
| 1LVA | 2V9V | N-terminal | a.4, a.4 | a.4,a.4 | 11 | 13 | **61; 73;** 64; 60 | S/R/D/E |
| 1OVT | 1NNT | N-terminal | c.94 | c.94 | 28 | 5 | **330;** 352 | R/D/E |
| 2QQI | 1KEX | N-terminal | b.18 | b.18 | 25 | 5 | **155**; 156 | D/E |
| 2WCP | 2WBX | N-terminal | b.18 | b.18 | 11 | 6 | **102**; 111 | D/E |
| 3M7D | 3M7G | N-terminal | a.265 | a.60 | 50 | 5 | **269**; 111 | R/D/E |
| 4C00 | 4BZA | N-terminal | d.319,d.319,d.319 | f.4 | 25 | 6 | **85; 64; 80;** 334 | S/R/D/E |

**^#^ Size of the domain (number of amino acids) in the order of domain arrangement in the protein with the common domain highlighted in bold.**

**^##^ Whether significant structural (S)/ residue-residue communication (R)/ dynamics (D) / energetics (E) differences are observed.**

**Table B.** SCOP fold annotations, length of the interface residues, length of the linker, size of the domain and the nature of differences observed for the homologous domain pair dataset.

| Single domain PDB id (SD) | Multi-domain PDB (MD) | Homologous domain directionality | SCOP fold (homologous domain) | SCOP fold (tethered domain) | No. of interface residues | Length of the linker | Size of the domain^*^ |
| --- | --- | --- | --- | --- | --- | --- | --- |
| 4WUI | 1PII | C-terminal | c.1 | c.1 | 36 | 3 | 254; **198** |
| 3ICH | 1IHG | N-terminal | b.62 | a.118 | 0 | 25 | **195**; 169 |
| 1SO7 | 1EUT | N-terminal | b.68 | b.1;b.18 | 20 | 5 | **358**; 103; 142 |
| 3B8A | 1HKC | Domain repeat | c.55; c.55 | c.55;c.55 | 9 | 17 | **207; 243; 205; 244** |

**^*^ Size of the domain (number of amino acids) in the order of domain arrangement in the protein with the homologous domain highlighted in bold.**

**Table C.** PDB codes and macromolecule name of pairs of proteins used for the analysis with their respective resolution (Å).

| PDB ID (MD) | PDB ID (ID) | Macromolecule Name | Resolution (Å)  (MD,ID) |
| --- | --- | --- | --- |
| 1B90 [[1](#_ENREF_1)] | 1CQY ** | β-amylase | 2.50, 1.90 |
| 1BLF [[2](#_ENREF_2)] | 1NKX [[3](#_ENREF_3)] | Lactoferrin | 2.80, 1.90 |
| 1BPD [[4](#_ENREF_4)] | 1RPL [[5](#_ENREF_5)] | DNA polymerase β | 3.60, 2.30 |
| 1C1Z [[6](#_ENREF_6)] | 4JHS ** | β-2-glycoprotein 1 | 2.87, 3.00 |
| 1CK7 [[7](#_ENREF_7)] | 1RTG [[8](#_ENREF_8)] | Gelatinase A | 2.80, 2.60 |
| 1EUT [[9](#_ENREF_9)] | 1EUR [[9](#_ENREF_9)] | Sialidase | 2.50, 1.82 |
| 1FCK [[10](#_ENREF_10)] | 1LCT [[11](#_ENREF_11)] | Lactoferrin | 2.20, 2.00 |
| 1FNF [[12](#_ENREF_12)] | 1FNA [[13](#_ENREF_13)] | Fibronectin | 2.00, 1.80 |
| 1GV2 ** | 1GV5 ** | MYB proto-oncogen protein | 1.68, 1.58 |
| 1LVA [[14](#_ENREF_14)] | 2V9V [[15](#_ENREF_15)] | Selenocysteine elongation factor | 2.12, 1.10 |
| 1OVT [[16](#_ENREF_16)] | 1NNT [[17](#_ENREF_17)] | Ovotransferrin | 2.40, 2.30 |
| 1T1E [[18](#_ENREF_18)] | 1GTG [[19](#_ENREF_19)] | Kumamolisin | 1.18, 2.30 |
| 1T4G [[20](#_ENREF_20)] | 2GDJ [[21](#_ENREF_21)] | DNA repair and recombination protein radA | 2.00, 2.50 |
| 1XTI [[22](#_ENREF_22)] | 1T5I [[23](#_ENREF_23)] | Probable ATP-dependent RNA helicase P47 | 1.95, 1.90 |
| 2QQI [[15](#_ENREF_15)] | 1KEX [[24](#_ENREF_24)] | Neuropilin-1 | 1.80, 1.90 |
| 2WCP [[25](#_ENREF_25)] | 2WBX [[25](#_ENREF_25)] | Cadherin-23 | 1.98, 1.50 |
| 3BTA [[26](#_ENREF_26)] | 3FUO [[27](#_ENREF_27)] | Botulinium neurotoxin type A | 3.20, 1.80 |
| 3M7D [[28](#_ENREF_28)] | 3M7G [[28](#_ENREF_28)] | Topoisomerase V | 1.82, 2.40 |
| 4C00 [[29](#_ENREF_29)] | 4BZA [[29](#_ENREF_29)] | Translocation and assembly module tama | 2.25, 1.84 |
| 4GCR [[30](#_ENREF_30)] | 1DSL [[31](#_ENREF_31)] | βγ-crystallin | 1.47, 1.55 |

** Crystal structure is yet to be published

**Table D.** PDB codes of homologous domains pairs with their resolution (Å).

| Macromolecule name | Single domain PDB id (SD) | Multi-domain PDB (MD) | Resolution (Å) (SD,MD) |
| --- | --- | --- | --- |
| Trp biosynthesis protein | 4WUI ** | 1PII [[32](#_ENREF_32)] | 1.09, 2.0 |
| Cyclophilin | 3ICH [[33](#_ENREF_33)] | 1IHG [[34](#_ENREF_34)] | 1.2, 1.8 |
| Sialidase | 1SO7 [[35](#_ENREF_35)] | 1EUT [[9](#_ENREF_9)] | 1.49, 2.5 |
| Hexokinase | 3B8A [[36](#_ENREF_36)] | 1HKC [[37](#_ENREF_37)] | 2.95, 2.8 |

** Crystal structure is yet to be published

**References**

1. Mikami B, Adachi M, Kage T, Sarikaya E, Nanmori T, Shinke R, et al. Structure of raw starch-digesting Bacillus cereus beta-amylase complexed with maltose. Biochemistry. 1999;38(22):7050-61. Epub 1999/06/03. doi: 10.1021/bi9829377. PubMed PMID: 10353816.

2. Moore SA, Anderson BF, Groom CR, Haridas M, Baker EN. Three-dimensional structure of diferric bovine lactoferrin at 2.8 A resolution. J Mol Biol. 1997;274(2):222-36. Epub 1998/02/12. doi: 10.1006/jmbi.1997.1386. PubMed PMID: 9398529.

3. Sharma S, Jasti J, Kumar J, Mohanty AK, Singh TP. Crystal structure of a proteolytically generated functional monoferric C-lobe of bovine lactoferrin at 1.9A resolution. J Mol Biol. 2003;331(2):485-96. Epub 2003/07/31. PubMed PMID: 12888354.

4. Sawaya MR, Pelletier H, Kumar A, Wilson SH, Kraut J. Crystal structure of rat DNA polymerase beta: evidence for a common polymerase mechanism. Science. 1994;264(5167):1930-5. Epub 1994/06/24. PubMed PMID: 7516581.

5. Davies JF, 2nd, Almassy RJ, Hostomska Z, Ferre RA, Hostomsky Z. 2.3 A crystal structure of the catalytic domain of DNA polymerase beta. Cell. 1994;76(6):1123-33. Epub 1994/03/25. PubMed PMID: 8137427.

6. Schwarzenbacher R, Zeth K, Diederichs K, Gries A, Kostner GM, Laggner P, et al. Crystal structure of human beta2-glycoprotein I: implications for phospholipid binding and the antiphospholipid syndrome. Embo J. 1999;18(22):6228-39. Epub 1999/11/24. doi: 10.1093/emboj/18.22.6228. PubMed PMID: 10562535; PubMed Central PMCID: PMC1171686.

7. Morgunova E, Tuuttila A, Bergmann U, Isupov M, Lindqvist Y, Schneider G, et al. Structure of human pro-matrix metalloproteinase-2: activation mechanism revealed. Science. 1999;284(5420):1667-70. Epub 1999/06/05. PubMed PMID: 10356396.

8. Gohlke U, Gomis-Ruth FX, Crabbe T, Murphy G, Docherty AJ, Bode W. The C-terminal (haemopexin-like) domain structure of human gelatinase A (MMP2): structural implications for its function. FEBS Lett. 1996;378(2):126-30. Epub 1996/01/08. PubMed PMID: 8549817.

9. Gaskell A, Crennell S, Taylor G. The three domains of a bacterial sialidase: a beta-propeller, an immunoglobulin module and a galactose-binding jelly-roll. Structure. 1995;3(11):1197-205. Epub 1995/11/15. PubMed PMID: 8591030.

10. Baker HM, Baker CJ, Smith CA, Baker EN. Metal substitution in transferrins: specific binding of cerium(IV) revealed by the crystal structure of cerium-substituted human lactoferrin. J Biol Inorg Chem. 2000;5(6):692-8. Epub 2000/12/29. PubMed PMID: 11128996.

11. Day CL, Anderson BF, Tweedie JW, Baker EN. Structure of the recombinant N-terminal lobe of human lactoferrin at 2.0 A resolution. J Mol Biol. 1993;232(4):1084-100. Epub 1993/08/20. doi: 10.1006/jmbi.1993.1462. PubMed PMID: 8371268.

12. Leahy DJ, Aukhil I, Erickson HP. 2.0 A crystal structure of a four-domain segment of human fibronectin encompassing the RGD loop and synergy region. Cell. 1996;84(1):155-64. Epub 1996/01/12. PubMed PMID: 8548820.

13. Dickinson CD, Veerapandian B, Dai XP, Hamlin RC, Xuong NH, Ruoslahti E, et al. Crystal structure of the tenth type III cell adhesion module of human fibronectin. J Mol Biol. 1994;236(4):1079-92. Epub 1994/03/04. PubMed PMID: 8120888.

14. Selmer M, Su XD. Crystal structure of an mRNA-binding fragment of Moorella thermoacetica elongation factor SelB. Embo J. 2002;21(15):4145-53. Epub 2002/07/30. PubMed PMID: 12145214; PubMed Central PMCID: PMC126154.

15. Appleton BA, Wu P, Maloney J, Yin J, Liang WC, Stawicki S, et al. Structural studies of neuropilin/antibody complexes provide insights into semaphorin and VEGF binding. Embo J. 2007;26(23):4902-12. Epub 2007/11/09. doi: 10.1038/sj.emboj.7601906. PubMed PMID: 17989695; PubMed Central PMCID: PMC2099469.

16. Kurokawa H, Mikami B, Hirose M. Crystal structure of diferric hen ovotransferrin at 2.4 A resolution. J Mol Biol. 1995;254(2):196-207. Epub 1995/11/24. doi: 10.1006/jmbi.1995.0611. PubMed PMID: 7490743.

17. Dewan JC, Mikami B, Hirose M, Sacchettini JC. Structural evidence for a pH-sensitive dilysine trigger in the hen ovotransferrin N-lobe: implications for transferrin iron release. Biochemistry. 1993;32(45):11963-8. Epub 1993/11/16. PubMed PMID: 8218271.

18. Comellas-Bigler M, Maskos K, Huber R, Oyama H, Oda K, Bode W. 1.2 A crystal structure of the serine carboxyl proteinase pro-kumamolisin; structure of an intact pro-subtilase. Structure. 2004;12(7):1313-23. Epub 2004/07/10. doi: 10.1016/j.str.2004.04.013. PubMed PMID: 15242607.

19. Comellas-Bigler M, Fuentes-Prior P, Maskos K, Huber R, Oyama H, Uchida K, et al. The 1.4 a crystal structure of kumamolysin: a thermostable serine-carboxyl-type proteinase. Structure. 2002;10(6):865-76. Epub 2002/06/12. PubMed PMID: 12057200.

20. Wu Y, He Y, Moya IA, Qian X, Luo Y. Crystal structure of archaeal recombinase RADA: a snapshot of its extended conformation. Mol Cell. 2004;15(3):423-35. Epub 2004/08/12. doi: 10.1016/j.molcel.2004.07.014. PubMed PMID: 15304222.

21. Galkin VE, Wu Y, Zhang XP, Qian X, He Y, Yu X, et al. The Rad51/RadA N-terminal domain activates nucleoprotein filament ATPase activity. Structure. 2006;14(6):983-92. Epub 2006/06/13. doi: 10.1016/j.str.2006.04.001. PubMed PMID: 16765891.

22. Shi H, Cordin O, Minder CM, Linder P, Xu RM. Crystal structure of the human ATP-dependent splicing and export factor UAP56. Proc Natl Acad Sci U S A. 2004;101(51):17628-33. Epub 2004/12/09. doi: 10.1073/pnas.0408172101. PubMed PMID: 15585580; PubMed Central PMCID: PMC539749.

23. Zhao R, Shen J, Green MR, MacMorris M, Blumenthal T. Crystal structure of UAP56, a DExD/H-box protein involved in pre-mRNA splicing and mRNA export. Structure. 2004;12(8):1373-81. Epub 2004/08/07. doi: 10.1016/j.str.2004.06.006. PubMed PMID: 15296731.

24. Lee CC, Kreusch A, McMullan D, Ng K, Spraggon G. Crystal structure of the human neuropilin-1 b1 domain. Structure. 2003;11(1):99-108. Epub 2003/01/09. PubMed PMID: 12517344.

25. Sotomayor M, Weihofen WA, Gaudet R, Corey DP. Structural determinants of cadherin-23 function in hearing and deafness. Neuron. 2010;66(1):85-100. Epub 2010/04/20. doi: 10.1016/j.neuron.2010.03.028. PubMed PMID: 20399731; PubMed Central PMCID: PMC2948466.

26. Lacy DB, Tepp W, Cohen AC, DasGupta BR, Stevens RC. Crystal structure of botulinum neurotoxin type A and implications for toxicity. Nat Struct Biol. 1998;5(10):898-902. Epub 1998/10/23. doi: 10.1038/2338. PubMed PMID: 9783750.

27. Fu Z, Chen C, Barbieri JT, Kim JJ, Baldwin MR. Glycosylated SV2 and gangliosides as dual receptors for botulinum neurotoxin serotype F. Biochemistry. 2009;48(24):5631-41. Epub 2009/05/30. doi: 10.1021/bi9002138. PubMed PMID: 19476346; PubMed Central PMCID: PMC2709598.

28. Rajan R, Taneja B, Mondragon A. Structures of minimal catalytic fragments of topoisomerase V reveals conformational changes relevant for DNA binding. Structure. 2010;18(7):829-38. Epub 2010/07/20. doi: 10.1016/j.str.2010.03.006. PubMed PMID: 20637419; PubMed Central PMCID: PMC2907367.

29. Gruss F, Zahringer F, Jakob RP, Burmann BM, Hiller S, Maier T. The structural basis of autotransporter translocation by TamA. Nat Struct Mol Biol. 2013;20(11):1318-20. Epub 2013/09/24. doi: 10.1038/nsmb.2689. PubMed PMID: 24056943.

30. Najmudin S, Nalini V, Driessen HP, Slingsby C, Blundell TL, Moss DS, et al. Structure of the bovine eye lens protein gammaB(gammaII)-crystallin at 1.47 A. Acta Crystallogr D Biol Crystallogr. 1993;49(Pt 2):223-33. Epub 1993/03/01. doi: 10.1107/S0907444992007601. PubMed PMID: 15299528.

31. Norledge BV, Mayr EM, Glockshuber R, Bateman OA, Slingsby C, Jaenicke R, et al. The X-ray structures of two mutant crystallin domains shed light on the evolution of multi-domain proteins. Nat Struct Biol. 1996;3(3):267-74. Epub 1996/03/01. PubMed PMID: 8605629.

32. Wilmanns M, Priestle JP, Niermann T, Jansonius JN. Three-dimensional structure of the bifunctional enzyme phosphoribosylanthranilate isomerase: indoleglycerolphosphate synthase from Escherichia coli refined at 2.0 A resolution. J Mol Biol. 1992;223(2):477-507. Epub 1992/01/20. PubMed PMID: 1738159.

33. Kozlov G, Bastos-Aristizabal S, Maattanen P, Rosenauer A, Zheng F, Killikelly A, et al. Structural basis of cyclophilin B binding by the calnexin/calreticulin P-domain. J Biol Chem. 2010;285(46):35551-7. Epub 2010/08/31. doi: 10.1074/jbc.M110.160101. PubMed PMID: 20801878; PubMed Central PMCID: PMC2975179.

34. Taylor P, Dornan J, Carrello A, Minchin RF, Ratajczak T, Walkinshaw MD. Two structures of cyclophilin 40: folding and fidelity in the TPR domains. Structure. 2001;9(5):431-8. Epub 2001/05/30. PubMed PMID: 11377203.

35. Chavas LM, Tringali C, Fusi P, Venerando B, Tettamanti G, Kato R, et al. Crystal structure of the human cytosolic sialidase Neu2. Evidence for the dynamic nature of substrate recognition. J Biol Chem. 2005;280(1):469-75. Epub 2004/10/27. doi: 10.1074/jbc.M411506200. PubMed PMID: 15501818.

36. Kuser P, Cupri F, Bleicher L, Polikarpov I. Crystal structure of yeast hexokinase PI in complex with glucose: A classical "induced fit" example revised. Proteins. 2008;72(2):731-40. Epub 2008/02/09. doi: 10.1002/prot.21956. PubMed PMID: 18260108.

37. Aleshin AE, Zeng C, Bartunik HD, Fromm HJ, Honzatko RB. Regulation of hexokinase I: crystal structure of recombinant human brain hexokinase complexed with glucose and phosphate. J Mol Biol. 1998;282(2):345-57. Epub 1998/09/15. doi: 10.1006/jmbi.1998.2017. PubMed PMID: 9735292.
